# Time-Restricted Eating for 12 Weeks Does Not Adversely Alter Bone Turnover in Overweight Adults

**DOI:** 10.3390/nu13041155

**Published:** 2021-03-31

**Authors:** Andrea J. Lobene, Satchidananda Panda, Douglas G. Mashek, Emily N. C. Manoogian, Kathleen M. Hill Gallant, Lisa S. Chow

**Affiliations:** 1 Department of Nutrition Science, Purdue University, West Lafayette, IN 47907, USA; hillkm@umn.edu; 2 Department of Kinesiology and Applied Physiology, University of Delaware, Newark, DE 19713, USA; 3 Salk Institute for Biological Studies, La Jolla, CA 92037, USA; panda@salk.edu (S.P.); emanoogian@salk.edu (E.N.C.M.); 4 Department of Biochemistry, Molecular Biology and Biophysics, University of Minnesota, Minneapolis, MN 55455, USA; dmashek@umn.edu; 5 Department of Medicine, Division of Diabetes, Endocrinology and Metabolism, University of Minnesota, Minneapolis, MN 55455, USA; chow0007@umn.edu; 6 Department of Food Science and Nutrition, University of Minnesota, St. Paul, MN 55108, USA

**Keywords:** time-restricted eating, bone turnover markers, bone health, humans

## Abstract

Weight loss is a major focus of research and public health efforts. Time-restricted eating (TRE) is shown to be effective for weight loss, but the impact on bone is unclear. Short-term TRE studies show no effect on bone mineral density (BMD), but no study has measured bone turnover markers. This secondary analysis examined the effect of 12 weeks of TRE vs. unrestricted eating on bone turnover and BMD. Overweight and obese adults aged 18–65 y (*n* = 20) were randomized to TRE (ad libitum 8-h eating window) or non-TRE. Serum N-terminal propeptide of type I collagen (P1NP), cross-linked N-telopeptide of type I collagen (NTX), and parathyroid hormone (PTH) levels were measured and dual-energy X-ray absorptiometry (DXA) scans were taken pre- and post-intervention. In both groups, P1NP decreased significantly (*p =* 0.04) but trended to a greater decrease in the non-TRE group (*p =* 0.07). The treatment time interaction for bone mineral content (BMC) was significant (*p =* 0.02), such that BMC increased in the TRE group and decreased in the non-TRE group. Change in P1NP was inversely correlated with change in weight (*p =* 0.04) overall, but not within each group. These findings suggest that TRE does not adversely affect bone over a moderate timeframe. Further research should examine the long-term effects of TRE on bone.

## 1. Introduction

Obesity rates have been rising over the past few decades, making it a major public health problem. Indeed, obesity affects 12% of adults globally [1] and 42.4% of adults in the U.S. [2] and contributes to death and disability due to a number of chronic diseases, including musculoskeletal disorders [1]. An often underappreciated effect of obesity is an increased risk for skeletal fracture [3,4]. Indeed, the majority of fractures occur in individuals who are overweight (body mass index (BMI) of 25.0–29.9 kg/m^2^) or obese (BMI ≥ 30 kg/m^2^) [5]. Having a higher body mass index (BMI) is associated with greater bone mineral density (BMD) [6,7,8], which, in part, can be attributed to increased mechanical loading [9]. However, evidence shows that obesity is associated with inferior bone quality [10] and poorer bone strength relative to body weight [11], as well as reduced physical function and increased incidence of falls [12]. Therefore, despite having greater bone mass, obesity is an important risk factor that leaves individuals vulnerable to fracture.

Because of the negative health outcomes related to obesity, weight loss has become a focus of significant research and public health efforts. While research has shown that weight loss is effective at reducing cardiometabolic risk factors [13], the effects of weight loss on bone are less conclusive. Weight loss tends to cause a decrease in BMD in older adults [14,15], but may have little or no effect on bone in younger adults, including pre-menopausal women [15,16]. In addition to the demographics of the study population, the type of weight loss intervention may determine the effect on bone. Indeed, including exercise as part of the weight loss intervention may help attenuate bone loss [17,18], with resistance training being particularly effective at preserving bone mass compared to aerobic exercise [18,19]. However, even with the addition of exercise, weight loss achieved through energy restriction may still result in bone loss in older adults [20]. These conflicting findings suggest that energy restriction may not be an appropriate weight loss strategy in all populations as it may lead to negative bone outcomes.

Weight loss can be achieved with a variety of interventions involving a restriction of energy intake [21]. However, recent research has explored the strategy of restricting the daily eating window to a consistent time of day rather than restricting energy intake, which is referred to as time-restricted eating (TRE). TRE involves limiting daily ad libitum food intake to a continuous 4–12 h period in order to align with the circadian rhythm [22,23]. Clinical studies have demonstrated that TRE is effective for weight loss [24,25,26,27] as well as improving body composition [27] and other cardiometabolic parameters [24,25,26,28]. However, the effects of TRE on bone health have not been thoroughly explored. To our knowledge, only two TRE studies have included bone outcomes. One study was a crossover study that compared 6 weeks of TRE to 6 weeks of normal feeding (no restrictions on timing or duration of eating) in healthy middle-aged and older adults [29]. The other was a parallel arm study that compared 12 weeks of TRE (from noon to 8:00 p.m.) to 12 weeks of consistent meal timing (three meals per day at normal meal times) in overweight adults aged 18–64 years [30]. Both studies found no differences in bone mineral density between the TRE group and the respective control group, which would suggest that TRE does not affect bone. However, no previous study has examined the effect of TRE on bone turnover markers, which change more rapidly in response to an intervention than BMD. Therefore, the aim of our study was to explore the effect of 12 weeks of TRE on bone mass and bone turnover compared to 12 weeks of usual daily intake (i.e., >14 h) in overweight and obese adults.

## 2. Materials and Methods

### 2.1. Study Design, Participants, and Intervention

This study was a secondary analysis of a feasibility study with the primary aim of examining the effects of TRE on weight loss, body composition, and metabolic parameters. The design and results of the parent study, as well as the Consolidated Standards of Reporting Trials (CONSORT) flow diagram, have been published previously [27]. In brief, this study was a randomized controlled trial with a parallel arm design. Inclusion and exclusion criteria are summarized in Table 1. Men and women aged 18–65 years with BMI ≥ 25 kg/m^2^ were recruited. Participants were screened and were considered eligible if they met the following criteria: had a stable work and sleep schedule, owned a smart phone, had a usual daily eating window ≥14 h, were consistently logging their eating occasions (≥2 eating events ≥5 h apart per day >1 day/week), were not pregnant or planning to become pregnant, and were free of chronic disease. Enrolled participants were randomly assigned to either the TRE or the non-TRE control group and were stratified by sex (male or female) and age (<45 years or ≥45 years old). Randomization assignments were determined in advance using the SAS pseudorandom number generator procedure. Participants in the TRE group were instructed to adhere to a self-selected daily 8-h eating window of ad libitum intake. Participants in the non-TRE group were instructed to continue their usual eating habits. No additional instructions or dietary education were provided to either group. The intervention lasted 12 weeks, and all participants were required to log their caloric intake using the myCircadianClock (mCC: www.mycircadianclock.org (accessed on 9 April 2020); Salk Institute, La Jolla, CA, USA) mobile application throughout the study period. Forty-seven participants were screened for eligibility, 22 began the intervention, and 2 were lost to follow-up, for a final analyzable sample of 20 participants. More details can be found in the published CONSORT diagram [27]. This study was registered on clinicaltrials.gov (NCT 03129581). All study procedures were approved by the University of Minnesota’s Institutional Review Board, and use of the mCC application was approved by the Salk Institutional Review Board. Participants provided written informed consent before beginning study procedures.

### 2.2. Data Collection and Bone Measures

The trial was conducted at the University of Minnesota (Minneapolis, MN, USA). Prior to beginning the intervention, baseline measurements were collected for all participants, including height, weight, blood draw (collected after at least an 8-h fast), and total-body dual-energy X-ray absorptiometry (DXA) scan (Lunar iDXA; GE Healthcare, Madison, WI, USA). At the end of the intervention period, the same measurements were collected. Pre-and post-intervention serum samples were used to measure markers of bone turnover in duplicate via ELISA, including parathyroid hormone (PTH; Quidel, San Diego, CA, USA; average coefficient of variation (CV) for duplicates, 12.5%), N-terminal propeptide of type I collagen (P1NP; Kendall Scientific, Lincolnshire, IL, USA; average CV for duplicates, 5.4%), a marker of bone formation, and cross-linked N-telopeptide of type I collagen (NTX; Kendall Scientific, Lincolnshire, IL, USA; average CV for duplicates, 8.0%), a marker of bone resorption. Pre- and post-intervention total body bone mineral content (BMC) and total body bone mineral density (BMD) were determined from the DXA scans using the enCore software (GE Healthcare, Chicago, IL, USA, version 16.2). All participants with complete data were included in the present analyses.

### 2.3. Statistical Analysis

Data were checked for normality and were found to be non-normally distributed and, thus, were log-transformed prior to analyses. Baseline differences in bone outcomes between groups were assessed using an independent samples *t*-test. The main effects of treatment, time, and the treatment-by-time interaction were assessed using a general linear mixed model (GLM). The relations between changes in BMI and weight and changes in bone outcomes were assessed in all participants and within each treatment group using Pearson bivariate correlations. All analyses were conducted using Statistical Analysis Software (SAS, version 9.4), and *p <* 0.05 was considered statistically significant.

## 3. Results

### 3.1. Baseline Characteristics and Changes in Weight

Twenty participants completed the study. Baseline characteristics relevant to the current analyses are shown in Table 2. All means are reported as mean *±* SEM unless otherwise indicated. More women participated in the study than men (*n* = 17 vs. *n* = 3). On average, participants were aged 45.5 *±* 2.7 years with a pre-intervention BMI of 34.1 *±* 1.7 kg/m^2^ and a usual daily eating window of 15.4 *±* 0.2 h. Data on the primary outcomes of the intervention have been published previously [27]. Briefly, the TRE group successfully reduced their daily eating window to 9.9 *±* 0.6 h per day. Body weight, fat mass, lean mass, and visceral fat were reduced in the TRE group compared to pre-intervention (−3.7% *±* 0.5; −4.0% *±* 0.9; −3.0 *±* 0.8, and −11.1% *±* 4.0, respectively), and changes in body weight, lean mass, and visceral fat were significant compared to the non-TRE group (all *p <* 0.05; data not shown).

### 3.2. Changes in Bone Turnover Markers

Baseline P1NP, NTX, and PTH levels were not significantly different between the TRE and non-TRE groups (*p =* 0.28, *p =* 0.20, and *p =* 0.13, respectively). Changes in bone turnover markers and the results of the GLM analyses are summarized in Table 3. For P1NP, the main effect of time was significant (*p =* 0.04), such that P1NP decreased in both groups. In addition, the treatment-by-time interaction approached significance (*p =* 0.07), such that the decrease in P1NP was larger in the non-TRE group than the TRE group (−4.07 *±* 1.4 ng/mL vs. −0.22 *±* 0.9 ng/mL). For both NTX and PTH, the main effects of treatment, time, and the treatment-by-time interaction were not significant (all *p >* 0.05). The results of the Pearson bivariate correlations are summarized in Table 3. When considering all participants, change in P1NP was negatively correlated with change in weight (r = −0.49, *p =* 0.04) and change in BMI (r = −0.52, *p =* 0.03). However, when examining the correlation between change in P1NP and changes in weight and BMI within both treatment groups, these correlations were no longer significant (all *p >* 0.05). In the non-TRE group, change in P1NP was positively correlated with change in eating window time (r = 0.71, *p =* 0.03). Changes in both NTX and PTH were not correlated with change in weight, changes in BMI, or change in eating window time.

### 3.3. Changes in Bone Mineral Content and Bone Mineral Density

Baseline BMC and BMD values were not significantly different between the TRE and non-TRE groups (*p =* 0.85 and *p =* 0.13, respectively). Changes in BMC and BMD and results of the GLM analyses are summarized in Table 3. For BMC, there was no significant main effect of treatment (*p =* 0.80) or time (*p =* 0.83), but the treatment-by-time interaction was significant (*p =* 0.02), such that mean BMC increased in the TRE group but decreased in the non-TRE group. However, for both groups, the changes in BMC were <20 g. For BMD, t-score, and z-score, there were no significant treatment effects, time effects, or treatment-by-time interactions (all *p >* 0.05). Table 4 summarizes the results of the Pearson bivariate correlations. In all participants, changes in BMC were not correlated with changes in weight (r = 0.02, *p =* 0.94) or changes in BMI (r = −0.06, *p =* 0.82). In all participants, change in BMD was not significantly correlated with changes in weight (r = 0.17, *p =* 0.48), changes in BMI (r = 0.08, *p =* 0.74), or changes in eating window time (r = −0.10, *p =* 0.68).

## 4. Discussion

This was the first study to compare the effects of 12 weeks of TRE versus 12 weeks of unrestricted (non-TRE) eating on markers of bone turnover. We observed a slight attenuation in the decrease in the bone formation marker P1NP with TRE compared to the larger decrease in P1NP in the non-TRE group, which could indicate a protective effect of TRE; however, this treatment-by-time interaction did not reach statistical significance. Regardless of treatment, change in P1NP was negatively correlated with change in weight and change in BMI. These findings suggest that TRE-associated weight loss may not adversely affect bone formation. In addition, there was a slight increase in BMC in the TRE group compared to a slight decrease in the non-TRE group, and this treatment-by-time interaction was significant. There were no significant findings with NTX or PTH. While this may suggest that TRE is beneficial for bone mass, it should be noted these changes were small (<20 g), and there were no significant findings with BMD. Our results suggest that, even though 12 weeks of TRE resulted in significant weight loss, lean mass loss, and fat loss compared to unrestricted eating [27], TRE did not adversely affect bone mass or bone turnover.

The prevalence of obesity has risen steadily over recent decades [2]. While having a greater body weight due to obesity is thought to increase BMD, evidence suggests that obesity is actually characterized by altered hormonal regulation of bone metabolism as well as poorer bone quality [31]. Implementing lifestyle and weight loss interventions has become a major focus of research and public health efforts, and evidence shows that lifestyle changes and weight loss are beneficial for improving cardiometabolic risk factors in this population [32]. However, the relationship between weight loss and bone is less clear, and the risks of various weight loss interventions to bone health should be thoroughly explored. Evidence suggests that diet-induced weight loss, achieved by restricting energy intake, can negatively affect bone [33], though the effects on bone may vary depending on the population of interest, the makeup of the diet, and the inclusion of exercise in the intervention [31]. A meta-analysis that examined the effects of diet-induced weight loss on bone found that interventions that lasted ≥6 months resulted in significant decreases in total hip BMD, but not interventions that were ≤3 months [34]. The study also reported no overall effect of diet-induced weight loss on lumbar spine BMD, and no effect on total body BMD, with the exception of a decrease in total body BMD with interventions lasting 6 months. This aligns with our current findings, which showed no effect of TRE on total-body BMD.

Bone turnover markers are a convenient way to examine dynamic and sub-clinical changes in bone in response to an intervention, such as diet-induced weight loss. The same meta-analysis discussed previously also examined changes in bone turnover markers and found no overall effect of diet-induced weight loss on bone formation markers (i.e., P1NP) but did find significant increases in bone resorption markers in studies lasting 2–3 months [34]. These findings are contrary to our current findings, which demonstrated no effects of 12 weeks of TRE on bone resorption. Importantly, the meta-analysis only included studies that utilized energy restriction, whereas our study did not restrict energy intake. This suggests that TRE, which allows unrestricted eating during the eating window, may be protective against bone resorption compared to other weight loss interventions that intentionally restrict energy intake.

The non-TRE group had higher levels of P1NP, NTX, and PTH at baseline, though these differences were not statistically significant (*p =* 0.28, *p =* 0.20, and *p =* 0.13, respectively). Given that participants were randomized to the TRE group or the non-TRE group, we assume that these slightly higher levels in the non-TRE group were due to chance. Importantly, we did not observe a significant treatment effect for any of these bone turnover markers. Thus, we do not believe that these slightly higher levels of P1NP, NTX, and PTH affected our primary outcome of interest, which was the treatment-by-time interaction. Interestingly, in the current analyses, despite the significant overall decrease in P1NP over time, we observed an inverse correlation between change in weight and change in P1NP (*p =* 0.04) in all participants, suggesting that greater weight loss is associated with a greater increase in P1NP. While this was unexpected, it is not unprecedented for weight loss interventions; observational studies of patients who underwent Roux-en-Y gastric bypass have shown them to have higher levels of P1NP one year [35] and ≥10 years [36] post-operatively, likely due to an overall increase in bone turnover. Notably, in our study, the changes in P1NP within each treatment group were not significant, and correlations between changes in P1NP and changes in weight were also not significant. Thus, this suggests that TRE has no effect on bone formation.

Unlike traditional diet-induced weight loss interventions, TRE involves restricting the daily period of intake, with no overt restriction on energy intake. While evidence suggests that TRE is effective for weight loss and improving cardiometabolic health in adults who are overweight or obese [24,25,27], few previous studies have explored the effect of TRE on bone. To our knowledge, only two short-term TRE studies have included bone outcomes, and both found no effect of TRE on BMD [29,30], which is consistent with our findings. However, these studies cannot provide conclusive evidence on the effect of TRE on bone, as it takes at least 1–2 years to see clinical changes in BMD as assessed by DXA [37,38]. Bone turnover markers, on the other hand, respond more rapidly to intervention and may provide better insight into the short-term effects on bone. While no previous study has explored the effects of TRE on bone turnover, studies have explored the short-term effects of other dietary interventions involving periods of fasting. Evidence suggests that alternate-day fasting, which is characterized by 24 h of severe energy restriction (~25% of energy needs) followed by 24 h of ad libitum intake, does not affect markers of bone turnover after 48 h [39] or 6 months [40] compared to usual intake, which is consistent with our findings. In addition, one study on Ramadan, which is a 1-month period in which Muslims fast from sunrise to sunset, found lower PTH levels secondary to increased calcium absorption and decreased serum phosphate [41], which suggests an overall benefit to bone. These findings indicate that dietary patterns that involve periods of fasting followed by ad libitum intake, at least assessed over the short term, may not adversely affect bone. Overall, this could suggest that weight loss interventions that alter the time period of eating, rather than the overall energy intake, are a more viable option for protecting bone health.

There are two potential confounders that may affect the relationship with diet-induced weight loss that we were unable to fully account for in our study: menopausal status and exercise. Menopause is characterized by a decrease in estrogen levels, resulting in bone loss [42,43], and evidence suggests that the effect of weight loss on bone varies by menopausal status [15]. Indeed, studies in premenopausal women have shown little to no change in bone mass with weight loss [16,44,45], while studies in postmenopausal women have shown significant decreases in bone mass with weight loss [46,47], which is not reversed with weight regain [48]. Our sample consisted mostly of women (85%) ranging in age from 25 to 63 years (mean 45 *±* 3.1 years), suggesting a heterogeneous sample of pre-, peri-, and postmenopausal status. Because of our small sample size and the higher number of women in our study relative to men, we are unable to explore sex differences, and we are unable to examine differences in treatment effects on bone mass and bone turnover by menopausal status. Exercise is another important factor in the relationship between weight loss and bone. Exercise helps increase and maintain bone mass directly via mechanical loading and indirectly by increasing muscle mass [49]. Indeed, studies show that exercise is effective at attenuating bone loss resulting from diet-induced weight loss [50,51], with resistance training shown to be more effective than aerobic exercise at preserving bone mass [19]. We did not collect specific data on exercise in the current study, which could be a mediating factor in the relationship between TRE and bone. However, the original paper did not report any differences in actigraphy-measured physical activity between the two groups (TRE vs. non-TRE) or relative to baseline [27].

Our study is strengthened by the randomized controlled trial design as well as the measurement of P1NP as a marker of bone formation as recommended by the International Osteoporosis Foundation and the International Federation of Clinical Chemistry and Laboratory Medicine [52]. However, it is not without limitations. In addition to our small sample size, our sample included few men and did not include older adults (aged >65 years), which precludes the generalizability of our findings to other populations. Although our results reflect total body BMD and BMC measurements, we acknowledge that not having scans of regional DXA areas clinically relevant to osteoporosis risk (e.g., lumbar spine and hip) is a limitation. Similarly, we also acknowledge that we do not have any information on smoking and alcohol use, which are two risk factors used in the calculation of 10-year fracture risk [53]. Because this was a secondary analysis, we were limited by the data collected as part of the parent study. Overall, our findings suggest that 12 weeks of TRE does not adversely affect bone turnover or bone mass. However, future studies should validate our findings in a larger sample as well as alternative populations, such as postmenopausal women, who may be at greater risk for bone loss. In addition, more research is needed to understand the long-term effects of TRE on bone, particularly on how TRE may affect bone geometry, bone quality, and overall fracture risk.

## Figures and Tables

**Table 1:** Summary of inclusion and exclusion criteria.

Inclusion Criteria:	Exclusion Criteria:
Ages 18–65 years BMI ≥ 25 kg/m^2^ Stable work and sleep schedule Owned a smart phone	Pregnancy/anticipated pregnancy Daily eating window < 14 h Uncontrolled disease/medical condition Incomplete logging of eating occasions

**Table 2:** Baseline characteristics.

Characteristic	All (*n* = 20)	TRE (*n* = 11)	Non-TRE (*n* = 9)
Age, y	45.5 *±* 2.7	46.5 *±* 3.7	44.2 *±* 4.1
BMI, kg/m^2^	34.1 *±* 1.7	33.8 *±* 2.3	34.4 *±* 2.6
Sex, *n*			
Women	17	9	8
Men	3	2	1
Daily eating window, h	15.4 *±* 0.2	15.2 *±* 0.2	15.6 *±* 0.4

Values represent mean *±* SEM unless otherwise indicated. BMI, body mass index; TRE, time-restricted eating.

**Table 3:** Summary of changes in bone outcomes.

	TRE (*n* = 11)			Non-TRE (*n* = 9)			*p*-Values		
Bone Outcome	Pre- Intervention	Post- Intervention	Change	Pre- Intervention	Post- Intervention	Change	Treatment	Time	Treatment × Time
Total body BMC, g	2852 *±* 173	2866 *±* 162	13.5 *±* 8.6	2801 *±* 159	2784 *±* 159	−17.8 *±* 7.9	0.80	0.83	0.02
Total body BMD, g/cm^2^	1.33 *±* 0.04	1.33 *±* 0.04	0.002 *±* 0.01	1.25 *±* 0.03	1.25 *±* 0.03	0.001 *±* 0.05	0.13	0.82	0.94
T-score, SD	2.2 *±* 0.3	2.2 *±* 0.3	0.02 *±* 0.04	1.5 *±* 0.3	1.5 *±* 0.3	0.01 *±* 0.04	0.08	0.56	0.73
Z-score, SD	1.6 *±* 0.3	1.8 *±* 0.4	0.14 *±* 0.1	0.6 *±* 0.3	0.7 *±* 0.3	0.06 *±* 0.06	0.61	0.10	0.95
P1NP, ng/mL ^1^	18.7 *±* 1.3	18.5 *±* 1.5	−0.22 *±* 0.9	28.8 *±* 8.8	24.7 *±* 8.2	−4.07 *±* 1.4	0.57	0.04	0.07
NTX, pg/mL ^1^	1074 *±* 112	1102 *±* 104	28.3 *±* 68	1780 *±* 513	1722 *±* 327	−57.9 *±* 237	0.13	0.30	0.83
PTH, pg/mL ^1^	45.0 *±* 6.9	52.3 *±* 5.6	7.3 *±* 3.3	62.8 *±* 8.2	75.2 *±* 10.1	12.3 *±* 13.5	0.09	0.10	0.64

Values represent mean *±* SEM unless otherwise indicated. *p*-values are based on the results of the general linear mixed model analyses. BMI, body mass index; BMC, bone mineral content; BMD, bone mineral density; P1NP, procollagen 1 N-terminal propeptide; NTX, cross-linked N-telopeptide of type I collagen; PTH, parathyroid hormone. ^1^ Analyses based on *n* = 9 participants from the TRE group who had pre- and post-intervention blood samples.

**Table 4:** Summary of Pearson bivariate correlations between change in weight and bone outcomes.

	Overall (*n* = 20)					
	Δ Weight		Δ BMI		Δ Eating window	
	r (95% CI)	*p*-value	r (95% CI)	*p*-value	r (95% CI)	*p*-value
Δ Total body BMC	0.02 (−0.43, 0.46)	0.94	−0.06 (−0.49, 0.82)	0.82	−0.40 (−0.71, 0.06)	0.08
Δ Total body BMD	0.17 (−0.30, 0.57)	0.48	0.08 (−0.38, 0.50)	0.74	−0.10 (−0.52, 0.36)	0.68
Δ P1NP	−0.49 (−0.77, −0.02)	0.04	−0.52 (−0.79, −0.05)	0.03	−0.29 (−0.66, 0.22)	0.26
Δ NTX	0.16 (−0.33, 0.58)	0.53	−0.03 (−0.49, 0.44)	0.90	−0.10 (−0.545, 0.39)	0.70
Δ PTH	0.004 (−0.46, 0.47)	0.99	−0.15 (−0.57, 0.35)	0.57	0.13 (−0.36, 0.56)	0.60
	**TRE (*n* = 11)**					
	**Δ Weight**		**Δ BMI**		**Δ Eating window**	
	**r (95% CI)**	***p*-value**	**r (95% CI)**	***p*-value**	**r (95% CI)**	***p*-value**
Δ Total body BMC	0.38 (−0.30, 0.79)	0.25	0.31 (−0.37, 0.76)	0.36	0.07 (−0.55, 0.64)	0.83
Δ Total body BMD	0.20 (−0.46, 0.71)	0.57	0.10 (−0.54, 0.66)	0.78	−0.14 (−0.68, 0.51)	0.69
Δ P1NP	−0.51 (−0.87, 0.26)	0.17	−0.31 (−0.80, 0.47)	0.44	0.04 (−0.64, 0.69)	0.92
Δ NTX	0.11 (−0.60, 0.72)	0.79	0.30 (−0.47, 0.80)	0.45	−0.26 (−0.78, 0.50)	0.52
Δ PTH	0.33 (−0.44, 0.81)	0.40	0.37 (−0.41, 0.82)	0.34	0.11 (−0.60, 0.72)	0.79
	**Non-TRE (*n* = 9)**					
	**Δ Weight**		**Δ BMI**		**Δ Eating window**	
	**r (95% CI)**	***p*-value**	**r (95% CI)**	***p*-value**	**r (95% CI)**	***p*-value**
Δ Total body BMC	0.31 (−0.46, 0.80)	0.43	0.34 (−0.44, 0.81)	0.39	0.10 (−0.61, 0.71)	0.80
Δ Total body BMD	0.31 (−0.46, 0.80)	0.43	0.19 (−0.55, 0.75)	0.63	−0.14 (−0.73, 0.58)	0.73
Δ P1NP	−0.26 (−0.78, 0.50)	0.52	−0.39 (−0.83, 0.37)	0.32	0.71 (0.04, 0.93)	0.03
Δ NTX	0.28 (−0.48, 0.79)	0.48	−0.06 (−0.70, 0.63)	0.88	0.03 (−0.65, 0.68)	0.93
Δ PTH	−0.13 (−0.73, 0.59)	0.76	−0.41 (−0.83, 0.37)	0.28	0.20 (−0.54, 0.76)	0.62

“Δ” or change values represent the mean of post-intervention values minus pre-intervention values. BMI, body mass index; BMC, bone mineral content; BMD, bone mineral density; P1NP, procollagen 1 N-terminal propeptide; NTX, cross-linked N-telopeptide of type I collagen; PTH, parathyroid hormone.

## Data Availability

These data will be available for future use by other investigators if proper IRB approval and material transfer agreements are obtained. Please contact the first author (A.J.L.) or the last author (L.S.C.) for details. These shared data will be provided de-identified for analysis.
